# A modified diet does not ameliorate muscle pathology in a mouse model for Duchenne muscular dystrophy

**DOI:** 10.1371/journal.pone.0215335

**Published:** 2019-04-24

**Authors:** Ingrid E. C. Verhaart, Davy van de Vijver, Joke W. Boertje-van der Meulen, Kayleigh Putker, Kevin Adamzek, Annemieke Aartsma-Rus, Maaike van Putten

**Affiliations:** Department of Human Genetics, Leiden University Medical Center, Leiden, the Netherlands; University of Sydney, AUSTRALIA

## Abstract

Duchenne muscular dystrophy (DMD) is caused by a lack of dystrophin protein. Next to direct effects on the muscles, this has also metabolic consequences. The influence of nutrition on disease progression becomes more and more recognized. Protein intake by DMD patients may be insufficient to meet the increased demand of the constantly regenerating muscle fibers. This led to the hypothesis that improving protein uptake by the muscles could have therapeutic effects. The present study examined the effects of a modified diet, which composition might stimulate muscle growth, on disease pathology in the D2-*mdx* mouse model. D2-*mdx* males were fed with either a control diet or modified diet, containing high amounts of branched-chain amino acids, vitamin D_3_ and ursolic acid, for six weeks. Our study indicates that the modified diet could not ameliorate the muscle pathology. No effects on bodyweight or weight of individual muscles were observed. Neither did the diet affect severity of fibrosis or calcification of the muscles.

## Introduction

Duchenne muscular dystrophy (DMD) is a severe muscle wasting disorder, affecting around 1 in 5 000 newborn boys [1]. First symptoms become apparent at approximately 2–3 years of age, whereupon muscle function gradually declines. Consequently, patients become wheelchair dependent around 10-12 years of age, followed by respiratory and cardiac failure, eventually leading to death around 30 years of age [2, 3]. DMD is caused by mutations in the *DMD* gene encoding for the dystrophin protein. Dystrophin plays an important role in stabilizing the muscle fibers and protecting them against damage during contractions. Its absence causes continues cycles of muscle degeneration and regeneration, eventually leading to the replacement of muscle tissue by adipose and fibrotic tissue [4].

The lack of dystrophin also has several metabolic consequences [5] and the impact and importance of healthy nutrition in DMD becomes more and more recognized [6]. One of the metabolic consequences are increased protein turnover and energy demands due to the continuous cycles of degradation and regeneration [7]. In DMD patients an imbalance in protein breakdown and synthesis is seen [8, 9]. This could partly be due to inadequate protein supply to the muscles, which led to the hypothesis that diet modification to enhance protein uptake may be beneficial in DMD.

Favorable effects of additional protein intake on muscle have been shown in other populations. Malnourished people, especially elderly where protein intake is insufficient, often suffer from sarcopenia. Here increased protein intake had positive effects on muscle synthesis and exercise performance [10, 11], indicating the possibility of influencing muscle metabolism by dietary intervention.

The effects of dietary protein intake in DMD patients and mouse models on disease pathology remain, however, unclear. Insufficient protein supply exaggerates the pathology in *mdx* mice [7]; however, little research has been done to assess whether this is also the case in humans. One study suggests that DMD patients do not require additional protein [12]. Studies on the effect of additional protein intake or supplementation of specific amino acids that may be beneficial for muscle health show mixed results. In *mdx* mice, a high protein diet did not show any effects [13]. Supplementation of specific amino acids (*i*.*e*. arginine, glutamine and glycine) seems beneficial in both patients and animal models [14–17]; however, in other studies no effect of the same amino acids was seen in DMD patients [18, 19]. In particular branched-chain amino acids (BCAA; leucine, isoleucine, and valine) have been shown to prevent muscle breakdown, stimulate protein synthesis and ameliorate disease pathology in mice [20] and patients [21, 22]. Besides supplementation of protein, vitamins and minerals are also important for muscle health [23]. For example, vitamin D_3_ and ursolic acid are shown to positively impact muscle strength and prevent muscle wasting [24–29].

In the majority of dietary intervention studies, C57BL/10ScSn-*Dmd*^*mdx*^/J (BL10-*mdx*) mice (*mdx* mice on a C57BL/10 background) were used, which is the most commonly used mouse model for DMD [30]. A drawback of this model is the relatively mild phenotype and disease progression compared to patients [31], making it more difficult to observe therapeutic effects. Recently, an *mdx* mouse model on a DBA/2J genetic background was generated (D2.B10-*Dmd*^*mdx*^/J; D2-*mdx*). D2-*mdx* mice have a more severe phenotype, lower muscle weight and increased amounts of fibrotic tissue, compared to the BL10-*mdx* mice [32–35]. These mice also show severe muscle atrophy and their regenerative capacity is impaired [36]. Muscle pathology is observed at young age. Taken together, this may make them a more suitable mouse model for DMD.

In the current study, we therefore utilized the more severely affected D2-*mdx* model to study the effect of a modified diet on muscle size and pathology. The composition of the diet we used has been based on nutraceuticals, *i*.*e*. BCAA, vitamin D_3_ and ursolic acid, that could be beneficial for muscle protein metabolism and exercise performance [37]. The aim of the study was to test whether this diet could attenuate or slow the disease progression in the D2-*mdx* mouse. We here show that our diet did not affect muscle weight or pathology.

## Materials and methods

### Mice and diets

All experiments were approved by the Animal Ethics Committee of the Leiden University Medical Center (permit 13211) and executed following EU-guidelines. Mice were bred by the animal facility of the Leiden University Medical Center and were housed in individually ventilated cages at 20.5°C with 12-hour dark-light cycles. D2.B10-*Dmd*^*mdx*^/J (D2-*mdx*) males were used for all experiments. Water was provided *ad libitum*. All efforts were made to minimize suffering.

Composition of standard RM3 chow (Special Diets Services; Essex, UK) has been described in Masuda *et al*. [38]. The modified diet powder consisted of 42% maltodextrin, 22.5% creamer; 10% whey protein concentrate; 9% calcium caseinate; 5.4% branched chain amino acids; 0.6% eriobotrya japonica extract containing 25% ursolic acid (RIA international; Germany); vitamins (amongst which 8.6 μg vitamin D_3_ per 100 g) and minerals (diet was manufactured by Aminolabs, Belgium). A gel was prepared consisting of 27% powder, 7.4% hydroxypropyl methylcellulose (HPMC; Sigma Aldrich; Zwijndrecht, the Netherlands) and mineral water.

### Study design

At five weeks of age D2-*mdx* males (n = 15) were fed standard RM3 chow (Special Diets Services) *ad libitum* to determine the average daily caloric intake. During this period food intake was weighed on a daily basis. At six weeks of age, mice were divided randomly into two experimental groups. Group sizes were based on an expected decrease of fibrosis in the diaphragm after treatment with the modified diet. To detect a decrease of 30% with 90% power the group size should be at least 6 mice per group. Group 1 (n = 7) was fed 100% RM3 chow. Group 2 (n = 8) was fed a modified diet, consisting of a mixture of RM3 chow and modified diet gel (1:1 caloric value). Caloric value and amount of protein in both diets was equal. Mice were provided with the amount of food determined during the preparation period plus an additional 20% to allow for extra nutrition during this growth period and to prevent insufficient food supply to the mice. If a mouse had finished all food on the next day, this amount was increased on a subsequent day to 25–35% to ensure that food intake was *ad libitum*. Bodyweight was measured twice a week. After six weeks, mice aged 12 weeks were sacrificed by cervical dislocation and muscles were isolated, weighted and snap frozen in 2-methylbutane (Sigma Aldrich) cooled in liquid nitrogen. The weights of the gastrocnemius, tibialis anterior, soleus and triceps muscles were normalized to bodyweight.

### Histology

Muscle sections of the gastrocnemius and the costal part of the diaphragm (8 μm thickness) were cut using a Leica CM3050 S Research Cryostat (Leica Microsystems B.V.; Amsterdam, the Netherlands) on whole muscle sections, in cross-section. Sections were collected on Superfrost Plus™ slides (Thermo Fisher Scientific; Menzel-Gläser; Germany).

For the quantification of fibrosis a Sirius Red staining was used. Slides were fixed in 4% paraformaldehyde for ten minutes, followed by 100% ethanol for five minutes. After air drying for 30 minutes, slides were rinsed in deionized water. Sections were stained with Sirius Red solution (Direct Red 80; Sigma-Aldrich) for 45 minutes, washed with 0.5% acetic acid water for five minutes and rinsed in deionized water. Thereafter, sections were dehydrated stepwise in ethanol (80%–90%–100%), incubated two times five minutes in xylene and mounted in Pertex (VWR International B.V; Amsterdam, the Netherlands). Pictures covering the entire cross-sectional area from the middle of the muscle were taken with the BZ-X700 microscope (Keyence; Osaka, Japan) at a ten times magnification and pictures were stitched using BZ-X700 analyzer software (Keyence). Background correction was performed using Adobe Photoshop CC 2018 (Adobe Systems Corporation; San Jose, CA, United States). Using ImageJ software (NIH), the percentage of fibrotic tissue was determined by dividing the Sirius Red positive area by the total tissue area. Analysis was performed by two to three blinded examiners and the average of their assessments was used.

To quantify calcification, sections were stained with Alizarin Red. Slides were fixed in acetone (Avantor; Arnhem, the Netherlands) for ten minutes, stained in Alizarin Red (Sigma Aldrich) for one minute, washed in acetone for 30 seconds and in acetone/xylene (1:1) for 15 seconds, incubated in xylene for 1.5 hours and mounted using Pertex (VWR International). The BZ-X700 microscope (Keyence) was used to take pictures along the entire cross-sectional area from the middle part of the muscle at a ten times magnification and pictures were stitched using BZ-X700 analyzer software (Keyence). Background correction was performed using Adobe Photoshop CC 2018 (Adobe Systems Corporation). Percentage of calcification was determined by dividing Alizarin Red positive areas by the total area using ImageJ software (NIH). Analyses were performed in a blinded manner by two to three examiners and the average of their assessments was used for analysis.

### Statistical analysis

Values are presented as means ± standard deviation (SD), except for bodyweight (measured over time), which is presented as mean ± standard error of the mean (SEM). R Studio version 1.1.453 [39] and Prism 7 (GraphPad Software Inc.; La Jolla, CA, USA) were used for statistical analysis. Bodyweights were compared fitting a linear mixed-effects model using treatment, age and treatment by age interaction as variables. A quadratic effect of age was included. Muscle weights, fibrosis and calcification data were normally distributed and were analyzed using a two-way ANOVA with Sidak’s multiple comparison test to correct for multiple testing. *P*<0.05 was considered significant.

## Results

Since protein supply may be insufficient in DMD and could exacerbate pathology, the effect of a modified diet on dystrophic pathology was examined in D2-*mdx* males. After determining caloric intake for one week, mice (aged six weeks) were randomized into two groups and fed with either a modified diet, rich in BCAA, vitamin D_3_ and ursolic acid (n = 8), or a control diet (RM3 chow) (n = 7) for a duration of six weeks. All mice were included in the analyses.

### Body and muscle weight

**Fig 1:**
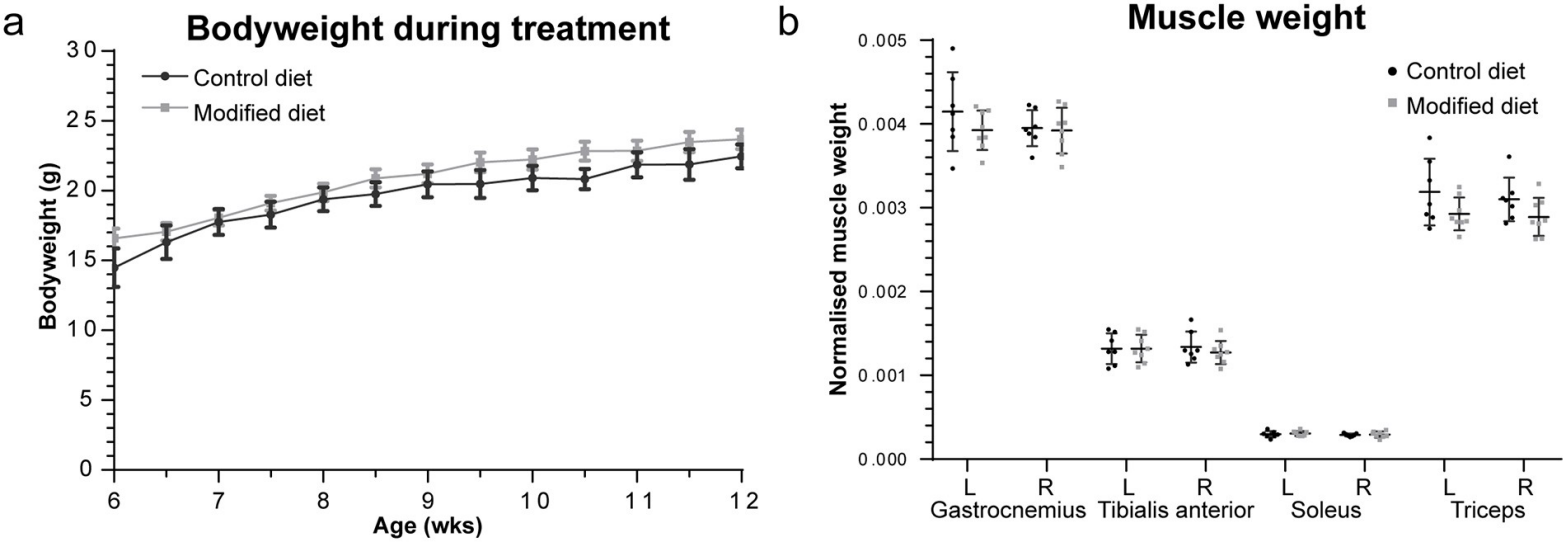
Body and muscle weight of mice during treatment with control or modified diet. (A) Average bodyweight of the mice during the experiment. Error bars represent SEM. (B) Weight of the muscles at sacrifice. Weights are normalized to bodyweight (g/g). Each dot represents an individual sample. Data are represented as mean ± SD.

No significant effect of the type of diet on bodyweight was observed (Fig 1A). Bodyweight was slightly higher in the mice fed with the modified diet from the study onset onwards. The growth curve was similar between both groups. Control mice gained on average 8.5 g ± 2.2 g, whereas treated mice gained 8.7 g ± 1.5 g (*P* = 0.85). In addition, no changes in weights of the individual muscles were observed after six weeks of treatment (Fig 1B).

### Fibrosis

**Fig 2:**
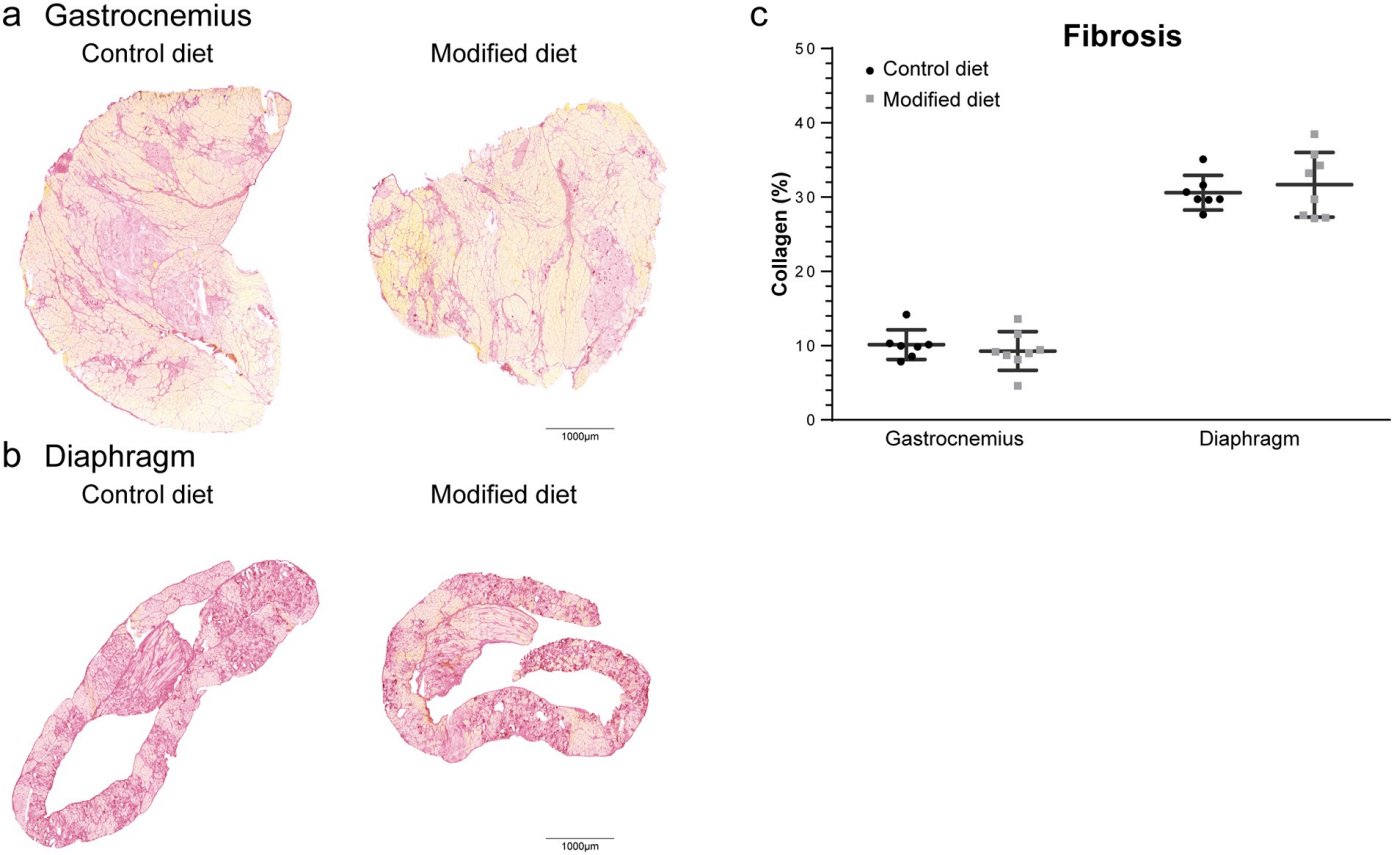
Effect of diet type on fibrosis. Representative pictures of fibrosis in (A) the gastrocnemius and (B) the diaphragm of control and modified diet fed D2-*mdx* males. Collagen (representative of fibrotic tissue) is stained red. (C) Quantification of fibrotic area. Each dot represents an individual sample. Data are represented as mean ± SD. Scale bars represent 1000 μm.

Fibrosis is a hallmark of DMD pathology. Therefore, the extent of collagen was quantified by Sirius Red staining of muscle cross-sections (Fig 2). In gastrocnemii of chow fed 12-week-old D2-*mdx* mice collagen levels of ~10% were observed. Quantities were not altered by the modified diet (Fig 2A and 2C). The diaphragm of BL10-*mdx* and D2*-mdx* mice is the most severely affected muscle, showing more prominent fibrosis [34, 40]. This was, however, not attenuated by the modified diet either (Fig 2B and 2C).

### Calcification

**Fig 3:**
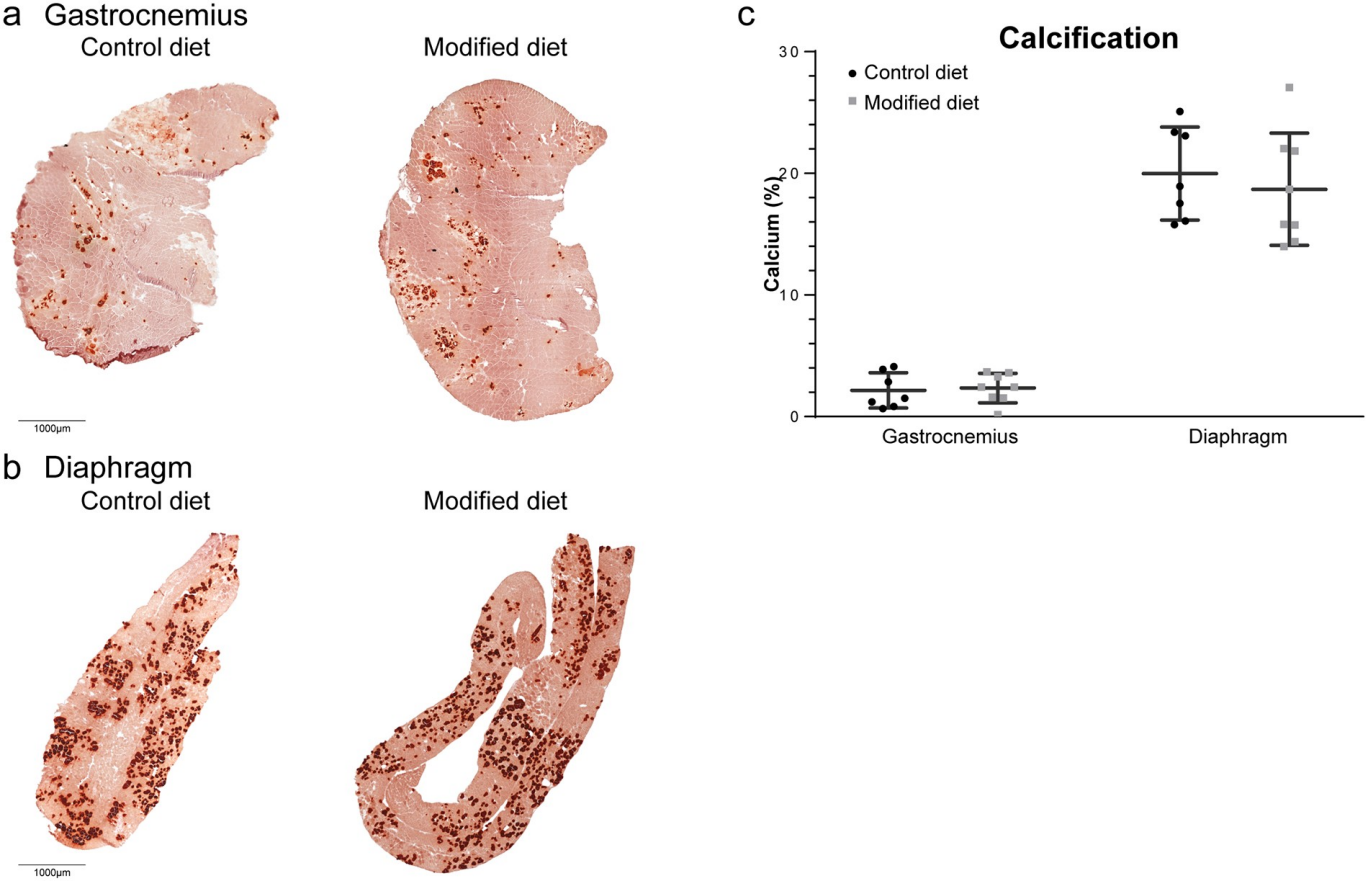
Effect of diet type on calcification. Representative pictures of calcification in (A) the gastrocnemius and (B) the diaphragm of control and modified diet fed D2-*mdx* males. Calcium deposits are stained red. (C) Quantification of calcified area. Each dot represents an individual sample. Data are represented as mean ± SD. Scale bars represent 1000 μm.

Large calcium deposits are observed in muscles of the D2-*mdx* mouse, which therefore could be a valuable readout for disease pathology [34]. No decrease in calcification was seen in the gastrocnemius of mice fed the modified diet (Fig 3A and 3C). As with fibrosis, especially the diaphragm is severely affected by calcification, showing levels up to 20% (Fig 3). However, the modified diet also had no effect on the amount of calcium deposits in the diaphragm (Fig 3B and 3C).

## Discussion

Next to direct effects of lack of dystrophin on muscle damage, many secondary consequences, like disturbances in muscle metabolism are seen in DMD patients [5]. The continuous muscle damage leads to increased degeneration and regeneration, which also impacts energy, especially protein, demands. Insufficient protein supply may therefore exaggerate muscle pathology. For BL10-*mdx* mice it is known that, especially in young mice, protein demand is very high due to rapid protein turnover and muscle hypertrophy [7]. It is not known whether this is also the case for D2-*mdx* mice as this model has not fully been characterized yet [34].

The D2-*mdx* mouse used in the current study is more severely affected compared to the BL10-*mdx* mouse [32, 33]. This may make it easier to observe effects; on the other hand it could also make it more difficult to reverse pathology by interventions with moderate effects. In the present study no indications were observed that modifying the diet in order to enhance protein uptake and prevent protein breakdown has beneficial effects on dystrophic muscle of D2-*mdx* mice. This result supports the conclusions of a previous study on protein supplementation in the BL10-*mdx* mouse model [13].

D2-*mdx* mice show severe muscle atrophy as evident by their low body and muscle weight and smaller fiber sizes [32]. The modified diet did not affect the bodyweight. The slightly higher bodyweight of the treated mice at study completion was due to a higher bodyweight at the start of the experiment. Comparison of the relative increase in bodyweight between both groups did not show any differences. The type of diet did not affect muscle weight; neither could it prevent the formation of fibrotic tissue. Another prominent feature of the D2-*mdx* mouse is calcification, which is only seen in minor extent in BL10-*mdx* mice [41] and not commonly observed in DMD patients. Heterotopic ossification contributes to the disease pathology [33], but could not be prevented by the modified diet.

Several factors may play a role in the lack of efficiency of the current study. Firstly, it could be due to the short duration of, and/or small sample size of the study. Larger sample sizes would increase the power of the study and enable detection of small effects, for example on bodyweight. The question is however, how relevant these small effects would be. The severity and rapid progression of the disease in patients requires larger effects to be clinically meaningful. Secondly, the current study was performed in young (six weeks old) mice. It may be that in older mice, where energy expenditure and protein synthesis are reduced compared to young mice [7], a modified diet could help to ameliorate the pathology. Thirdly, the extra protein available to the muscle may still not be sufficient to compensate for the increased protein demand of the muscles. The amount of BCAA supplied via the diet was, however, comparable to the BCAA supplementation in the previous study, where beneficial effects were observed in *mdx* mice (~1.5 mg/g bodyweight) [20, 42]. Moreover, it is not sure whether DMD patients require extra protein [12, 43]. There are some indications that DMD patients require more dietary proteins [44]. Several studies suggest that in young DMD patients, protein intake is higher compared to their peers, while this is lower in adolescent and adult patients [44–46]. In healthy young children, increased protein intake has been associated with increased BMI and risk of obesity [47–49]. Obesity is often seen in young DMD patients, due to the use of corticosteroids, increased energy intake and decreased activity [50, 51]. This leads to increased load on the muscle and can worsen disease pathology. Older patients, however, are at risk of being underweight [52]. Therefore, effects of protein supplementation may differ between age groups and may be more beneficial for adult patients. This however, requires more research to understand the protein requirements and consequences of dietary composition for DMD patients of different ages.

In conclusion, our modified diet could not ameliorate the disease pathology in D2-*mdx* mice.

## Supporting information

S1 Dataset (XLSX)
